# Thia-Michael Reaction: The Route to Promising Covalent Adaptable Networks

**DOI:** 10.3390/polym14204457

**Published:** 2022-10-21

**Authors:** Dimitri Berne, Vincent Ladmiral, Eric Leclerc, Sylvain Caillol

**Affiliations:** ICGM, Univ Montpellier, CNRS, ENSCM, 34090 Montpellier, France

**Keywords:** Michael, vitrimers, covalent adaptable network, thia-Michael

## Abstract

While the Michael addition has been employed for more than 130 years for the synthesis of a vast diversity of compounds, the reversibility of this reaction when heteronucleophiles are involved has been generally less considered. First applied to medicinal chemistry, the reversible character of the hetero-Michael reactions has recently been explored for the synthesis of Covalent Adaptable Networks (CANs), in particular the thia-Michael reaction and more recently the aza-Michael reaction. In these cross-linked networks, exchange reactions take place between two Michael adducts by successive dissociation and association steps. In order to understand and precisely control the exchange in these CANs, it is necessary to get an insight into the critical parameters influencing the Michael addition and the dissociation rates of Michael adducts by reconsidering previous studies on these matters. This review presents the progress in the understanding of the thia-Michael reaction over the years as well as the latest developments and plausible future directions to prepare CANs based on this reaction. The potential of aza-Michael reaction for CANs application is highlighted in a specific section with comparison with thia-Michael-based CANs.

## 1. Introduction

The Michael addition was named after Arthur Michael (1853–1942) [1] who discovered the addition reaction of sodiomalonate esters or sodioacetoacetate esters onto α,β-unsaturated esters [2]. This reaction corresponds to a 1,4-conjugate addition of a stabilized carbanion onto an electron-poor double bond such as α,β-unsaturated carbonyl compounds. Other Michael additions based on the addition of an anion to activated alkenes have been further developed by using other nucleophiles. Thia-, oxa- and aza-Michael reactions correspond, respectively, to the polar 1,4-addition of a thiol, an alcohol and an amine (Michael donor) onto double bonds activated by a conjugated electron-withdrawing group (Michael acceptor) [3]. In contrast to the historical Michael reaction, these “hetero-Michael” additions are potentially reversible under appropriate conditions.

Covalent adaptable networks (CANs) have gained a lot of interest from the scientific community, as this polymer family represents a potentially sustainable solution for the reduction of polymer wastes. Indeed, thermosets, which were originally designed to be dimensionally stable and provide high mechanical and chemical resistance, cannot be easily reprocessed [4]. CANs are composed of a 3D network structure in which some covalent bonds show reversibility upon thermal stimulus, conferring self-healing, shape memory and reprocessing properties to the material [5]. The first CANs, composed of disulfide bonds and silyl ethers, have been developed decades ago [6,7] and recently re-evaluated [8,9]. Numerous exchange or reversible reactions such as Diels–Alder cycloaddition [10,11,12,13], transesterification [14,15,16,17], transcarbamoylation [18,19,20,21] or transamidation [22,23] have been employed to design and prepare CANs.

The exchange reactions are generally classified according to their mechanism, either associative or dissociative [24]. Based on this mechanistic difference, vitrimers would correspond to CANs having an associative exchange mechanism, whereas the exchange reaction will follow a dissociative mechanism in dissociative CANs. An associative exchange ensures a constant cross-link density, whereas a dissociative one generates a temporary loss of network connectivity when the exchange takes place. Therefore, only associative networks should show an Arrhenius behavior and therefore be considered as vitrimers (associative CANs). Nevertheless, as highlighted by Dichtel and Elling [25], some dissociative CANs based on oxime-enabled transcarbamoylation [26] and *trans-N*-alkylation [27] for instance also showed this typical behavior. Hence, the major difference between associative and dissociative CANs is often a decrease of the storage modulus with temperature as observed in different studies on dissociative CANs [22,28]. 

Several review articles on CANs and vitrimers have been published in the last few years, giving an overview of the chemistry and physics of these materials [24,29,30,31] or focusing on specific exchange reactions such as the transesterification [32] or transcarbamoylation [33] for example. In this context, the Michael/retro-Michael equilibrium appears as a new promising exchange reaction for CAN applications which has not yet been reviewed, to the best of our knowledge. Hence, this review highlights the main parameters influencing the Michael reaction rate and reversibility, and present an overview of the Michael reaction-based CANs. This review is mainly focusing on the thia-Michael as this reaction was the most evaluated one for CANs application. In comparison, aza-Michael based CANs (described in Section 4.) have been less evaluated but represent a promising way for further CAN development. 

## 2. Thia-Michael Reaction

**Scheme 1:**
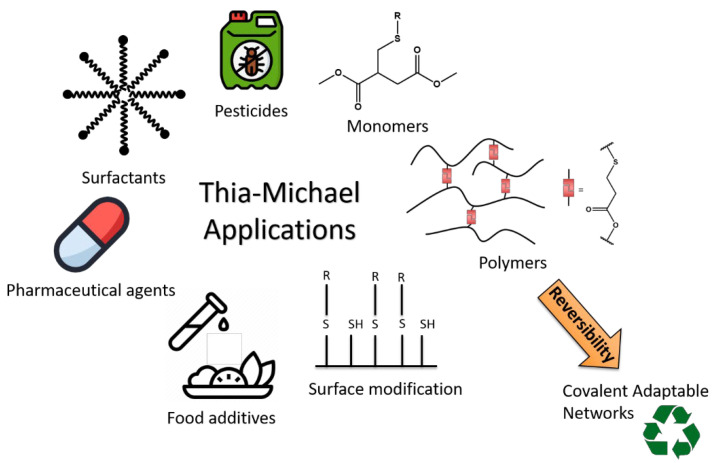
Application fields of thia-Michael addition.

The thia-Michael addition is an intensively used reaction in industry for the synthesis of food additives and surfactants, pesticides and pharmaceutical agents [34,35,36,37,38]. In recent decades, the thia-Michael addition has found a new field of application in materials science. Indeed, the simplicity of this addition reaction enables to easily synthesize a large range of monomers [39] or dendrimers [40] and can also be used to perform surface modification [41]. Polymer networks synthesized via thia-Michael addition have been also explored. For instance, thio-acrylate networks were prepared by using a thia-Michael addition followed by the photopolymerization of an excess of acrylate functional groups [42]. Using a similar idea, A. Lowe et al. performed sequential phosphine-catalyzed thia-Michael addition followed by radical-mediated thiol−yne reaction to prepare polyfunctional materials [43]. The thia-Michael addition has also been used to cross-link acrylated epoxidized soybean oil in order to obtain partially bio-based networks [44]. Recently, some dynamic covalent networks (CANs) have been designed on the basis of the reversibility of the thia-Michael addition (Scheme 1) [45,46]. For the following discussion, the term “thia-Michael addition” refers only to the addition step, whereas the term “thia-Michael equilibrium” refers to the overall thia-Michael reaction (elimination/addition). Finally, the term “thia-Michael exchange” refers to the reaction between a Michael adduct and a Michael donor or acceptor. The thia-Michael exchange, which is focused on in this review, is of course dependent on a few critical parameters such as the promotor or catalyst, the solvent and the structure of the reagents (thiol and Michael acceptor). 

**Scheme 2:**
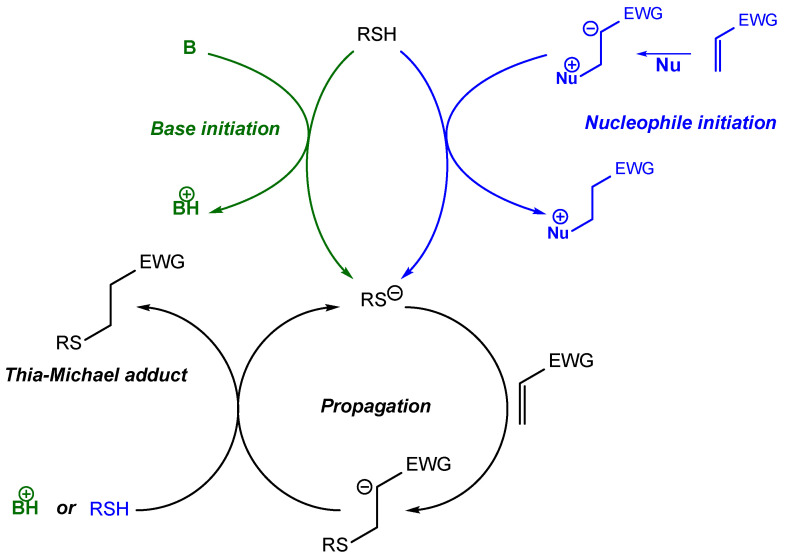
The mechanism of the base-catalyzed and nucleophile-initiated thia-Michael additions between a thiol and a vinyl group.

*Influence of the promotor/catalyst*: Thia-Michael additions are generally catalyzed with weak Brønsted bases such as triethylamine or by Lewis bases such as phosphines (nucleophile-initiation) [47,48]. Bowman et al., studied these two mechanisms [49] and established that, in both cases, the addition takes place in three steps. First, a thiolate is formed by reaction of the thiol either with the base or with an anion generated from the nucleophilic catalyst and the Michael acceptor. Then, this thiolate adds to the acceptor to generate a negatively charged addition product. Finally, a proton exchange occurs between this addition product and either a new thiol function or the protonated base to form the final Michael adduct (Scheme 2) [49]. For the nucleophile-initiated thia-Michael addition, a zwitterionic intermediate is generally formed from the attack of the Lewis base onto the Michael acceptor. Subsequently, the thiol is deprotonated by the zwitterionic intermediate leading to the formation of the thiolate anion and a phosphonium ester. After addition of the thiolate onto the Michael acceptor, a proton exchange occurs with a new thiol, leading to a new thiolate and therefore to a propagation of the reaction. In this case, the nucleophile is only used as an initiator of the reaction. For the base-catalyzed mechanism, the thiolate anion is obtained in one step by acid-base reaction. In this case, the base and the unreacted thiol can both perform proton exchange with the negatively charged addition product, whereas in the nucleophile-initiated thia-Michael addition only the thiol species participate to this exchange. The base-catalyzed rate is negatively impacted by the presence of the protonated base in the system as it slows down the formation of new thiolate. Therefore, the nucleophile-initiated thia-Michael addition generally proceeds faster and requires lower catalyst loadings compared to the base-catalyzed pathway [50].

*Influence of the solvent*: The solvent used for a chemical reaction is generally chosen to solubilize the reactants and catalysts and to minimize side reactions [3]. However, solvent characteristics also play a role in the reaction kinetics. For instance, the thiol-acrylate reaction catalyzed by triethylamine was accelerated in a polar medium such as dimethyl sulfoxide (DMSO), propylene carbonate and N-ethyl urethane compared to the neat reaction [51]. Du Prez et al. investigated the Michael addition of thiol with maleimide, acrylate and vinyl sulfone and the NEt_3_-catalyzed thiol–iso(thio)cyanate reactions in DMF and in chloroform [52]. Overall, they observed higher reaction rates in polar aprotic solvents such as DMF and DMSO, which favor the formation of thiolates through a stabilization of this negatively charged species due to their high dielectric constants [53]. Therefore, solvents promoting the formation of thiolates (initiation) should be chosen to promote the thia-Michael addition. This effect was particularly visible in the case of base-catalyzed reactions for which initial deprotonation is the limiting step of the mechanism. Similarly, addition reaction rates are increased in high pH solution as thiolate anions are easily formed under these conditions [54].

*Influence of the substrate structures*: The thiol basicity and the electron deficiency of the Michael acceptor as well as the steric hindrance of the reactants influence the thia-Michael addition. The influence of the bulkiness of the substituents has been largely studied for the aza- and carbon-Michael additions, leading to the intuitive conclusion that the reaction rate decreases as the α- and β-substituents size increases [55,56]. Recently, Bowman et al., highlighted a similar behavior for thia-Michael additions with alkyl thiol. Indeed, when the steric hindrance of the thiol increases, the addition rate decreases. However, when mercaptopropionates were used, the addition of a methyl substituent in α-position of the thiol increased slightly the reaction rate, leading to the opposite conclusion [57]. Nonetheless, as previously reported, the rate-limiting step is different between the addition of alkyl thiols or mercaptopropionates [58]. Due to their respective basicity and nucleophilic characters, the addition of alkyl thiols is rate-limited by the proton exchange step whereas the mercaptopropionate reactions are limited by the thiolate addition step. Thus, the decrease of the deprotonation rate induced by the higher electron-density of substituted alkyl thiols, added to their increased steric hindrance, results in a reduction of the overall reaction rate [59]. In contrast, the addition rate of mercaptopropionates is directly dependent on the thiol nucleophilicity and the most-electron-rich thiolate (α-methylmercatopropionate) adds faster onto the double bond. In this study, it was also demonstrated through a comparison between vinyl sulfone, fumarate and acrylate that the more electron-deficient the acceptor, the higher the addition rate. Tirelli et al. also showed that thia-Michael addition rate was higher for acrylates than for acrylamides, thus highlighting the electronegativity dependence of this reaction [60].

The thia-Michael addition is highly efficient, rapid and more selective than the radical-mediated thiol-ene reactions [61]. Indeed, the thiol-ene reaction involves the formation of a thiyl radical that directly adds onto the carbon-carbon double bond, yielding a carbon-centered radical intermediate. In a second step, an hydrogen transfer occurs with a second thiol molecule to form the thiol-ene addition product and a new thiyl radical [53]. Secondary products are mainly formed through the homopolymerization of alkene radicals generated during the reaction [62,63]. In contrast to the radical-initiated thiol-ene reaction, the thia-Michael addition can be performed under neat conditions and at low temperature, with a higher tolerance to functionality [50]. 

In summary, the thia-Michael addition can be considered as a performant “click” reaction that can be used in organic synthesis, surface modification and polymer chemistry [64].

## 3. Thia-Michael Equilibrium and Exchange Kinetics

**Scheme 3:**
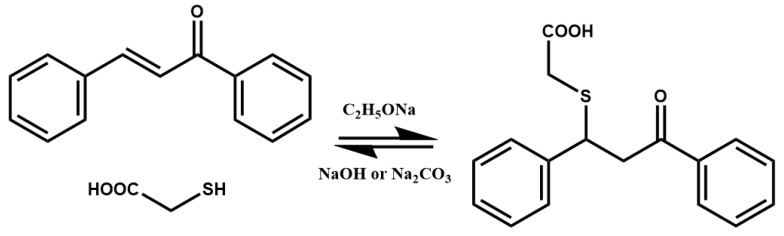
Thia-Michael equilibrium of thioglycolic acid on benzalacetophenone.

The reversible character of the thia-Michael addition was evidenced for the first time in 1931 by B. Nicolet [65] who demonstrated the reversibility of the addition of thioglycolic acid on benzalacetophenone (Scheme 3). The thia-Michael adducts dissociated when placed in a basic medium composed of sodium hydroxide or sodium carbonate. 

**Scheme 4:**
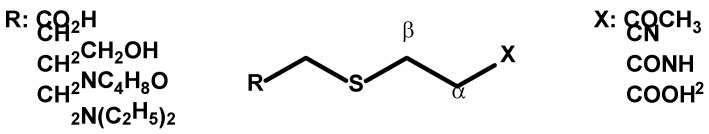
Thia-Michael adducts.

The dissociation phenomenon was then specifically evaluated by C. Allen and W. Humphlett [66,67]. The dissociation of thia-Michael adducts was monitored 2 min after dissolution in water or in ethanol with and without sodium hydroxide at different temperatures. The influence of the groups present on the acceptor or on the thiol was also examined in these articles (Scheme 4). They demonstrated that the elimination reaction was favored as the electron withdrawing ability of the X group increased by enhancing the acidity of the α-hydrogen of the alkene function, as suggested by the order of dissociation observed in this study (ketone > nitrile > amide > acid). In contrast, the correlation between the structure of the thiol and the thia-Michael reversibility was however not obvious from these studies. 

**Scheme 5:**
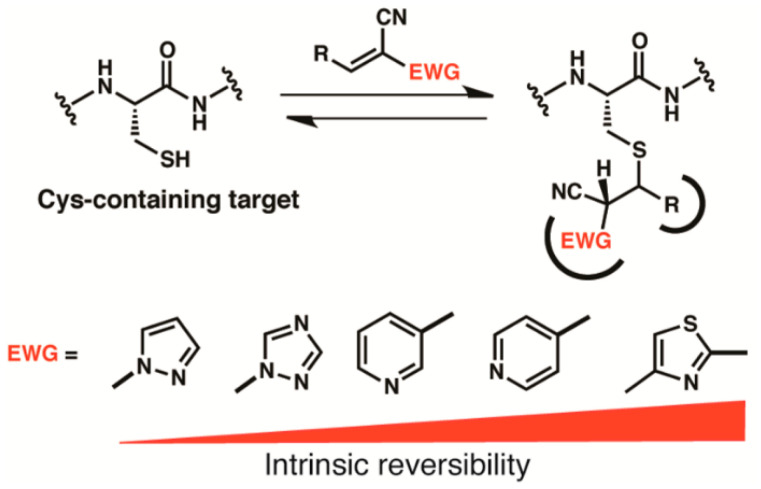
Reversibility of dually activated α-cyano Michael acceptors (Adapted from Ref. [72], copyright 2022, with permission of American Chemical Society).

In 1968, Holmes et al. [68] observed the fast reaction of dually activated Michael acceptors with butanethiol. In this study, a cyano group is associated with another electron-withdrawing group on the α-position of the acceptor, leading to a fast addition reaction. However, the thia-Michael adducts could not be isolated due to the high reversibility of the reaction [69,70]. The dynamic character of the thia-Michael equilibrium is therefore largely influenced by the EWG placed in the α-position. This perspective has notably found applications in medicinal chemistry. The thia-Michael equilibrium indeed served as an application of the concept of reversible covalent inhibition, enabling the development of protein inhibitors with long-lasting, but not permanent action [71]. This principle was specifically introduced by Taunton et al. who designed reversible Michael acceptors as cysteine-targeting inhibitors [70,72]. They highlighted the influence of a second EWG on α-cyano Michael acceptor with kinetic and computational analyses of the thia-Michael equilibrium (Scheme 5). The presence of a methylthiazole group placed in the α-position of the acrylonitrile had a strong promoting impact on the reversibility of the thia-Michael addition, similar to the one observed with an α-amide group. This study also demonstrated that the presence of a phenyl group at the β-position did not have a significant effect on the reaction reversibility.

In 2016, Houk et al. also investigated the influence of an additional α-EWG and of a β-substituent by DFT calculations [73]. They demonstrated that an α-EWG lowers the energy barrier for the thiol addition but also makes the addition reaction thermodynamically less favorable. The presence of an aryl or of a branched alkyl group on the β-carbon led to a rapidly reversible thia-Michael addition. The equilibrium of the thiol addition with benzalcyanoacetate-based Michael acceptors was specifically assessed by Herbert et al. [74]. The equilibrium constants were determined at room temperature for a series of benzalcyanoacetate and were highly dependent of the electron-withdrawing/-donating ability of the substituent placed on the *para*-position of the β-phenyl ring. Hence, as the electron-withdrawing ability increased, the equilibrium shifted preferentially to the associated state. By performing NMR in temperature, the authors demonstrated also that the equilibrium shifted to the dissociative state as the temperature increased.

**Scheme 6:**
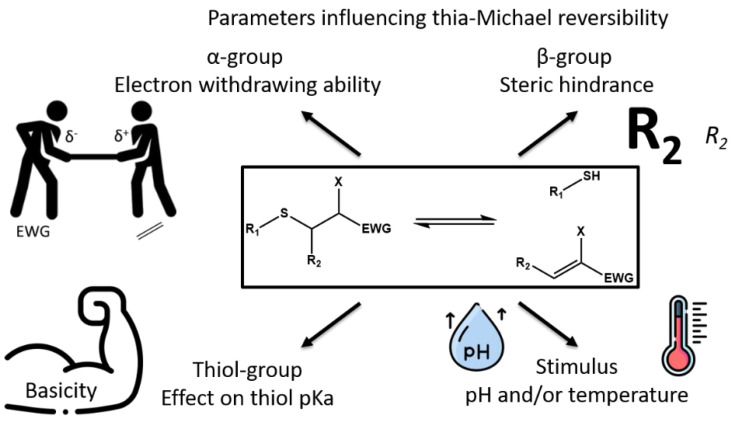
Parameters influencing thia-Michael equilibrium applications.

In conclusion of these first studies, three major parameters must be considered to control thia-Michael equilibrium: the α-EWG group, the stimulus used to activate the retro thia-Michael, and to a lesser extent the β-carbon substituents (Scheme 6). Indeed, even if the thermal stimulus is the most common one, increase of the pH value or the presence of a base in the reaction medium also favor the retro-thia-Michael addition. For instance, thermodynamic and kinetic analyses of the thia-Michael equilibrium of glutathione onto helenalin derivatives were performed at different pH values [75,76]. It was demonstrated that the addition reaction proceeded rapidly and was reversible at basic pH, and that oppositely the reaction was slow and irreversible at low pH. The Michael addition of thiols onto 5-methylene pyrrolones has also been described as reversible in an alkali buffer (pH 9.5) and exchange reaction with other thiols was observed at 7.5 pH [77].

**Scheme 7:**
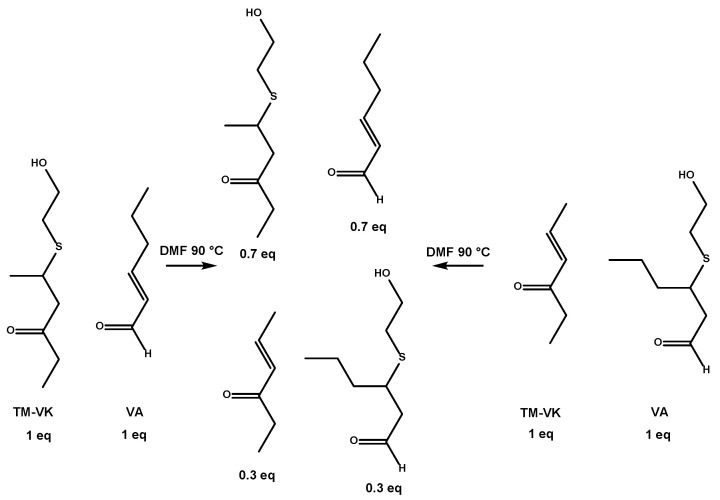
Thia-Michael exchange between 4-hexen-3-one (VK) and 2-hexen-1-al (VA) and their adducts with 2-mercapoethanol (TM-VK and TM-VA, respectively).

The existence of the thia-Michael equilibrium offers the possibility to use this reaction in dynamic exchange chemistry. Indeed, under appropriate conditions, the thia-Michael adduct dissociates and the free thiol generated can react onto another acceptor present in the system. Some articles describe the use of the thia-Michael addition/elimination as the exchange reaction at work in CANs and highlighted the dynamic character of the reaction via kinetic analyses on model molecules. Konkolewicz et al. in a first article on this topic published in 2016 [45], studied the reversibility of the thia-Michael addition on ketone-, acrylate- and aldehyde-derived thia-Michael adducts, respectively TM-VK, TM-HEA and TM-VA. At 90 °C, the evolution over time of a DMF solution of a thia-Michael adduct with an exchangeable acceptor was monitored by NMR. Equilibrium composition was independent of the starting materials, for instance, TM-VK/VA (1:1) mixture reached the same equilibrium (VA/TM-VA = 70/30) as TM-VA/VK (1:1) mixture, which confirms the presence of a dynamic equilibrium between thia-Michael adducts (Scheme 7). This kinetic study also allowed to confirm a stability order [66]. Indeed, TM-HEA was the most stable adduct followed by TM-VA while the less stable adduct was TM-VK. 

Chakma et al. studied the dynamic exchange of *N*-methylmaleimide thia-Michael adducts at 90 °C in DMF by NMR kinetic analyses [78]. In this study, two thiols were in competition for the addition onto *N*-methylmaleimide. The equilibrium was reached after 8 h and composed of approximately 50% of the thio-phenol adduct (TP-MM) and 50% of the 2-mercaptoethanol adduct (ME-MM), regardless of the starting mixtures (TP mm exchanging with free 2-mercapoethanol (ME) or ME mm exchanging with free thiophenol (TP)). There was therefore negligible energy difference between these two adducts, confirming the previous statement that the thiol substituent has only a minor impact on the adduct stability. This dynamic exchange was used to prepare degradable hydrogels containing reversible maleimide thia-Michael adduct [79,80]. In the presence of a competitive thiol (glutathione), the exchange reaction took place and degraded the hydrogels, permitting the release of an encapsulated compound. 

**Scheme 8:**
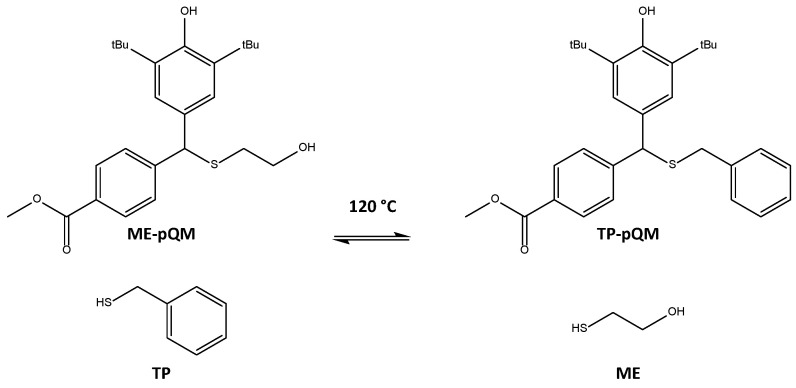
Thia-Michael equilibrium between thiophenol (TP)/ 2-mercaptoethanol-pQM adduct (ME-pQM) and mercaptoethanol (ME)/ thiophenol-pQM adduct (TP-pQM).

Zhang et al. studied the 1,6-conjugated addition exchange between 2,6-di-*tert*-butyl-7-phenyl-p-quinone methide (pQM) and thiol nucleophiles [81]. The dynamic character of this vinylogous Michael reaction was evaluated via kinetic analyses of a mixture of a pQM thia-Michael adduct with a competitive thiol (Scheme 8). At room temperature, the pQM thia-Michael adduct was stable in the presence of another free thiol. However, when a thermal stimulus (120 °C) was applied to this mixture, the thia-Michael exchange reaction took place. After 4 h of reaction, an equilibrium state–once again independent on the starting materials—composed of 37% of thiophenol-pQM adduct (TP-pQM) and 63% of 2-mercaptoethanol-pQM adduct (ME-pQM) was reached. This study thus demonstrated the existence of a dynamic equilibrium for this 1,6-conjugated thiol addition.

The thia-Michael reversibility has been explored under different conditions and for different kind of thia-Michael adducts, providing insight into the thia-Michael dynamic exchange. Nevertheless, it should be noted that kinetic exchange studies were mainly performed in the presence of a competitive thiol. It could be thus interesting for future studies to look at the exchange of a thia-Michael adduct with a Michael acceptor and a Michael donor. Indeed, these data could bring more information about the reactions involved in polymer networks. Moreover, a systematic study of not only the reversibility but also the thia-Michael exchange rate according to the nature of the substituents on the Michael acceptors and donors would be of great help. In conclusion, despite some remaining grey areas in the knowledge of the Michael exchange, these results are promising enough to consider thia-Michael exchange as a valuable reaction for dynamic material chemistry application. 

## 4. Thia-Michael Exchange in CANs

**Scheme 9:**
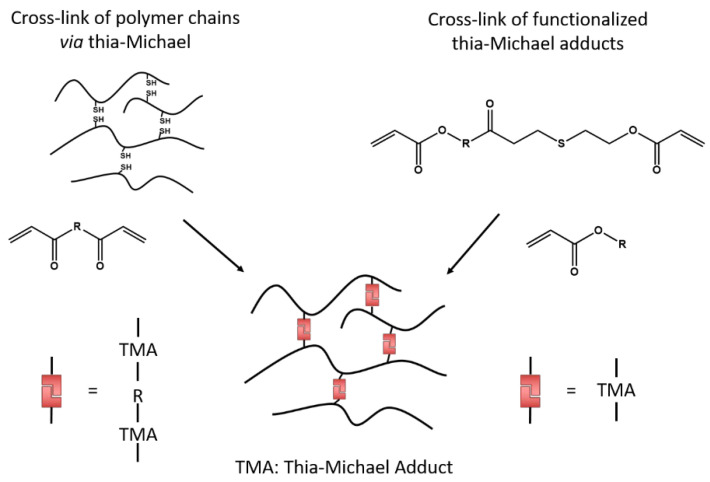
Two synthesis ways of thia-Michael CANs.

Two main methods of synthesis have been developed to insert thia-Michael adducts in CANs (Scheme 9). In the first method, the thia-Michael addition was used as the cross-linking reaction. For instance, linear polymer chains possessing pendant thiols functions and prepared by RAFT polymerization were crosslinked by reaction with a di-maleimide in dioxane at room temperature [78]. Miller et al., used the thia-Michael base-catalyzed addition as the cross-linking reaction, with a tetravalent thiol-terminated PEG added onto a tetravalent cyano-acrylate-terminated PEG to form a CAN [82]. The second method consists of using monomers already containing reversible thia-Michael adducts to form a 3D network. The most common approach in that case was to synthesize di-acrylate monomers by thia-Michael addition and copolymerize them with mono-functional acrylates by conventional radical polymerization leading to the formation of a cross-linked network [45,46,83,84]. Another approach used a pQM thia-Michael adduct terminated with two alcohol functions which was copolymerized with other diols and triols by reaction with a diisocyanate [81].

**Figure 1:**
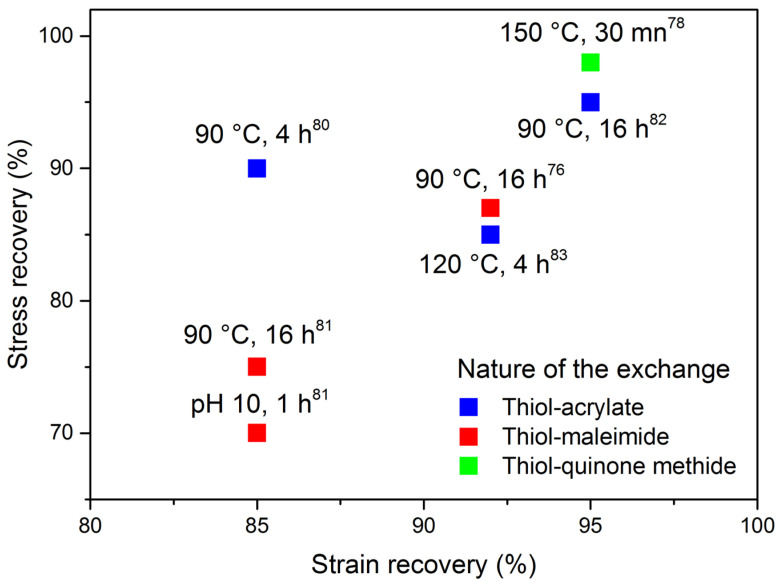
Strain and recovery percentages according to the nature of the exchange and the reprocessing conditions.

Stress/strain experiment is the usual test employed to test the reshapability of thia-Michael based CANs. The stress and strain at break are measured on an initial sample, which is then cut in two pieces, self-healed by pH or thermal treatment before being re-analyzed. The sample is considered as healed when a high strain and stress recovery is observed and when the break occurs at a different place that the initial cut [84]. The temperature of curing is usually kept under 100 °C, as it was previously demonstrated by Raman spectroscopy that free thiol are thermally unstable (elimination of H_2_S) above this temperature [85]. The conditions of curing reported in the various studies published so far are shown in Figure 1.

Rheological experiments (creep, stress relaxations and frequency sweep) were also performed to evaluate the flow of the material. Surprisingly, for thia-Michael-based CAN studies, only few articles report stress relaxation or creep experiments at different temperatures whereas, as mentioned in the introduction, even if the thia-Michael exchange follows a dissociative mechanism, an Arrhenius behavior could be observed. For instance, complete relaxation data on thiol-quinone methide CAN allowed the determination of an activation energy of 86 kJ.mol^−1^ for this specific exchange [81]. In addition, creep and relaxation experiments performed at room temperature demonstrated that the synthesized materials were mechanically stable at this temperature and that almost no exchange occurred under these conditions [46,78]. 

**Figure 2:**
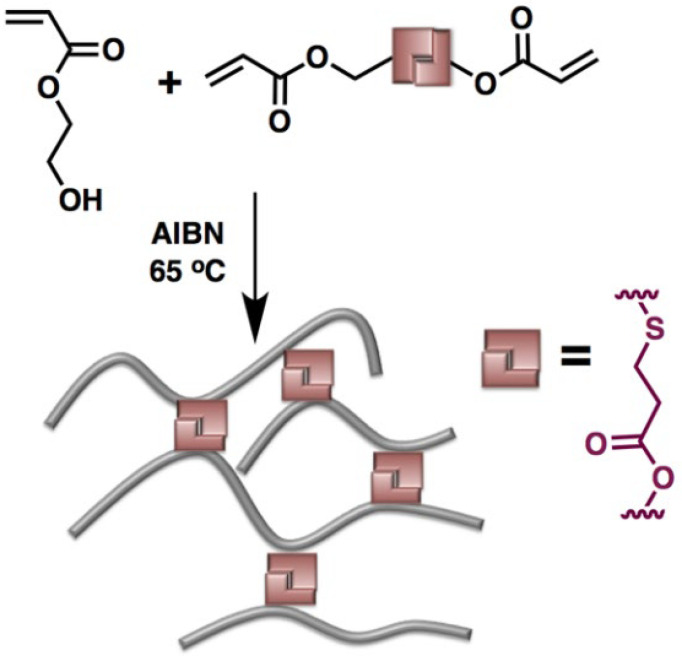
Example of a CAN based on thia-Michael exchange. (Adapted from Ref. [45], copyright 2022, with permission of American Chemical Society).

Some observations can be made on these studies. Konkolewicz et al., noticed that the self-healing properties were reduced when the material cross-link density increased. Two hypothesis were proposed to explain this observation [45]. First, as it was also noticed on polymers synthesized by RAFT polymerization [78], an increase of cross-linking could lead to a decrease of chain mobility, inducing a reduction of the material reprocessing ability. The second hypothesis is related to the specific composition of this network (Figure 2): indeed, for a lower cross-link density, more free alcohol wase present in the network. According to the authors, the presence of these hydroxyl functions could facilitate self-healing because of existing supramolecular interactions between the two materials parts during reprocessing [45]. The time/temperature dependence observed for some of the presented CANs can also be highlighted. Indeed, similar recovery results were obtained for samples treated at 100 °C for 48 h and samples treated at 120 °C for 8 h [84]. Finally, it can be noted that thia-Michael exchange can be coupled with another exchange reaction. For instance, heat and light responsive materials were synthesized by coupling thia-Michael acrylate adducts with coumarin adducts [83] which can undergo photoreversible dimerization [86].

The existence of an equilibrium for benzalcyanoacetate-adducts at room temperature inhibits the formation of a fully cured network at room temperature. However, Herbert et al. demonstrated that, by tuning the electronic characteristics of the substituent placed on the *para*-position of the β-phenyl ring, it was possible to modify the final conversion of the thia-Michael addition [74]. Hence, in presence of nitro groups, thiols functions were converted up to 92% whereas in presence of methoxy groups the conversion was only of 24%. The extent of thia-Michael linkages influences directly the mechanical properties of the initial material. Under thermal stimulus, the dissociation of the thia-Michael linkages can reach such an extent that, above a temperature which depends on the group used, the system loses connectivity and flows. Such materials can find applications as pressure-sensitive/hot melt adhesives [87]. 

The thia-Michal exchange is therefore a promising reaction for the development of CANs, as the exchange rate can be tuned by introducing groups with specific electronic characteristics. Moreover, the insertion of dynamic thia-Michael linkages can easily be performed since the thia-Michael addition constitutes an efficient polymerization reaction. The applicability of these CANs has not yet been thoroughly assessed but materials of this type could be used for composite preparation [88]. Indeed, a resin composed of thia-Michael bond could be depolymerized in the presence of an adequate Michael donor/acceptor. Moreover, the thia-Michael click chemistry could be adapted to the preparation of films which could demonstrate healing behavior under the appropriate stimulus.

## 5. Perspectives on the Development of Aza-Michael Exchange Reactions in CANs

This review initially aimed at highlighting the recent advances made on thia-Michael exchange in CANs. However, Du Prez et al., recently demonstrated that aza-Michael exchange was also possible under thermal stimulus [89]. Just like the thia-Michael exchange, the reversible character of the aza-Michael reaction was first highlighted on model molecules. This catalyst-free exchange was studied by reacting the addition product of *N*-methylbutylamine onto 2-ethylhexyl acrylate with a different acrylate (2-ethylhexyl 3-(buty)methylamino)propanoate) in DMF at temperature ranging from 100 to 160 °C. It was observed that aza-Michael exchange rate was relatively slow compared to transesterification. The authors claimed that it is necessary to reach a high temperature to observe the dissociation of the aza-Michael adduct. This preliminary kinetic analysis was followed by the synthesis of CANs by mixing commercial di-amines and multifunctional acrylates. The reprocessing temperature (180 °C) of these CANs was much higher than the one used in CANs based on thiol-acrylate exchange, indicating a higher stability of the C–N bond compared to the C–S one. One noticeable difference between thia and aza-Michael CANs results from the Michael addition itself. Indeed, the aza-Michael can occur twice in the presence of a primary amine function leading to the formation of a tertiary amine which can then catalyze the exchange reactions in the network (including the Michael exchanges). 

**Scheme 10:**
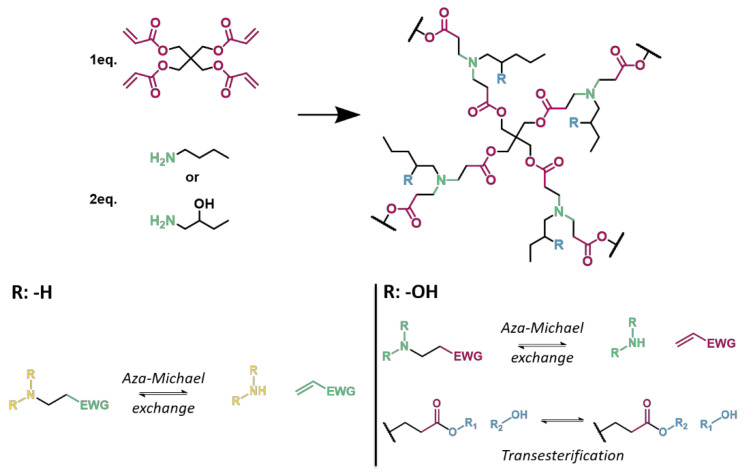
CANs using either only aza-Michael exchange or a dual aza-Michael exchange/ transesterification.

The combination of aza-Michael exchange with transesterification was also evaluated in this initial article [89]. This work was then extended by our group in a study showing the enhanced reactivity of a β-hydroxyamine and its potential for the synthesis of dual covalent adaptable networks [90]. In these networks the presence of ester and hydroxyl functions allowed to perform transesterification as well as aza-Michael exchange. The possibility of having these concomitant exchange reactions enables to access CANs with lower reshaping temperature compared to their non-hydroxylated counterparts in which only an aza-Michael exchange could take place (Scheme 10). The combination of two exchange reactions was also recently performed by synthesizing CANs based on aza-Michael and vinylogous urethane exchange [91]. Coupling the aza-Michael exchange with a faster exchange reaction is one way to improve the reprocessing properties of these materials. The combination of multiple exchange reactions could also be a concept to develop with the thia-Michael exchange, especially since exchangeable disulphide bonds can be easily obtained from the thiol function used for the thia-Michael exchange. 

Regarding the development of CANs only based on aza-Michael exchange, the possibility of promoting this exchange thanks to an adequately placed, strongly electronegative group, was appealing. Accordingly, it has been demonstrated at the molecular and material scale that the introduction of a CF_3_ group in α-position of the ester enabled to highly improve the aza-Michael exchange rate. Indeed, the inductive effects of the CF_3_ group activates both the dissociation of the β-amino ester and the Michael addition of another amine onto the acceptor [92].

These recent results on dynamic thia- and aza-Michael, shed a new light on many thermoset materials which could be re-evaluated as CANs or for which depolymerization or recycling pathway could be investigated. Indeed, thermosets mentioned in the introduction [42,43] and featuring thia-Michael linkages could potentially show similar reshaping and depolymerization properties as the materials described in the core of this review [76,78,80,81,82,83]. Similarly, materials initially defined as thermosets synthesized by aza-Michael could be revisited as CANs [93]. For instance, thermosets prepared from bio-based resources by aza-Michael addition of amine cross-linkers on acrylated epoxidized sucrose soyate [94], linseed oil [95], soybean or olive oils [96] could show dynamic properties under thermal stimulus. Likewise, materials obtained by cross-linking of amine-terminated poly(hydroxyurethane) with multifunctional acrylates could behave as CANs under appropriate stimulus [97]. 

In conclusion, the introduction of the concept of exchange chemistry for thia- and aza-Michael is a breakthrough in polymer chemistry as materials initially classified as thermosets may easily gain reprocessing/recycling properties under appropriate conditions. This work paves the way for a further and wider application of Michael exchange reaction in materials chemistry. 

## Data Availability

Not applicable.
